# Recent Advances in the Understanding of Stress Resistance Mechanisms in Probiotics: Relevance for the Design of Functional Food Systems

**DOI:** 10.1007/s12602-024-10273-9

**Published:** 2024-06-03

**Authors:** Ana Yanina Bustos, María Pía Taranto, Carla Luciana Gerez, Sofia Agriopoulou, Slim Smaoui, Theodoros Varzakas, Hesham Ali El Enshasy

**Affiliations:** 1 Centro de Investigación en Biofísica Aplicada y Alimentos (CIBAAL/UNSE-CONICET), RN 9-Km 1125, (4206), Santiago del Estero, Argentina; 2 Facultad de Agronomía y Agroindustrias (FAyA), Universidad Nacional de Santiago del Estero, Av. Belgrano Sur 1912, (4200), Santiago del Estero, Argentina; 3 Facultad de Humanidades, Ciencias Sociales y de La Salud (FHU), Universidad Nacional de Santiago del Estero, Av. Belgrano Sur 1912, (4200), Santiago del Estero, Argentina; 4 Centro de Referencia Para Lactobacilos (CONICET-CERELA), Chacabuco 145, (4000), San Miguel de Tucumán, Argentina; 5 Department of Food Science and Technology, University of the Peloponnese, 24100 Antikalamos Messinia, Kalamata, Greece; 6 Laboratory of Microbial Biotechnology and Engineering Enzymes (LMBEE), Center of Biotechnology of Sfax (CBS), University of Sfax, Road of Sidi Mansour Km 6, P.O. Box 1177, 3018 Sfax, Tunisia; 7 Institute of Bioproduct Development (IBD), Universiti Teknologi Malaysia (UTM), 81310 Johor, Malaysia; 8 Faculty of Chemical and Energy Engineering, Universiti Teknologi Malaysia (UTM), 81310 Johor, Malaysia; 9 City of Scientific Research and Technology Applications (SRTA), New Borg Al Arab, 21934 Egypt

**Keywords:** Probiotics, Functional foods, Stress conditions, Gastrointestinal passage, Postbiotics

## Abstract

In recent years, more and more scientific community, food producers, and food industry show increased interest in functional foods containing probiotics, which is a big challenge. The consumption of probiotics in the context of a balanced diet through the consumption of functional foods or through the intake of pharmaceutical preparations has proven to contribute to the improvement of human health, even contributing to the prevention of diseases. In order for probiotics to be considered suitable for consumption, they must contain a minimum concentration of viable cells, namely, at least 10^7^ colony forming units of beneficial microbes per gram. Ensuring the viability of bacterial cells until the moment of consumption is the overriding priority of functional probiotic food manufacturers. Probiotic bacteria are subject to stress conditions not only during food manufacturing but also during gastrointestinal passage, which limit or even compromise their functionality. This paper first examines all the stressful conditions faced by probiotic cells in their production stages and related to the conditions present in the bioreactor fermentation and drying processes as well as factors related to the food matrix and storage. The stress situations faced by probiotic microorganisms during the gastrointestinal transit especially during stomach and intestinal residence are also analyzed. In order to understand the adaptation mechanisms of probiotic bacteria to gastrointestinal stress, intrinsic and adaptive mechanisms identified in probiotic strains in response to acid stress and to bile and bile acid stress are analyzed. In addition, improvement strategies for multiple stress tolerance of lactic acid bacteria through directions dealing with stress, accumulation of metabolites, use of protectants, and regulation of technological parameters are examined. Finally, the definition of postbiotics, inanimate microorganisms and/or their components conferring health benefits, is also introduced. Postbiotics include cell lysates, enzymes, and cell wall fragments derived from probiotic bacteria and may represent an alternative to the use of probiotics, when they do not tolerate stressful conditions.

## Introduction

In recent years, the usefulness of the human microbiome for both short-term and long-term human health has been clarified by the scientific community. The microbiome significantly determines the development of the immune system. The role of probiotics has received much scientific attention and is the subject of research in recent decades [1]. Probiotics are living microorganisms that act through competitive exclusion to protect the host from various pathogens and provide nutrients from the breakdown of indigestible dietary carbohydrates, [2, 3] e.g., increasing short-chain fatty acid (SCFA) production [4]. Moreover, symptoms from acute infectious diarrhea, antibiotic-associated diarrhea, *Clostridium difficile*-associated diarrhea, ulcerative colitis, and irritable bowel syndrome could be treated with probiotics [5]. The administration of probiotics requires either a medical prescription or not, and their composition includes microorganisms that have similarities with the common bacteria found in the gut, most often *Bifidobacterium* and *Lactobacillus* spp. that produce lactic acid [5–7]. Control of commercial probiotic products can be characterized as insufficient since probiotics are branded and not characterized by the bacterial strain and formulations or manufacturing protocols may be subject to changes over time, dramatically affecting their effectiveness [5].

Although there is an inextricable link between the biological activity of probiotics and the beneficial effects on human health through the consumption of foods containing them, this benefit can be degraded due to the stressful conditions faced by the microorganisms from the moment they are produced, until the incorporation in food, their subsequent storage, and consumption [8]. Stress situations faced by probiotic microorganisms during food production may concern the conditions of the fermentation carried out in the bioreactor and preparation of microbial stocks, the drying processes, such as, spray drying and freeze-drying, the food matrix, as well as their storage and characteristics of food [8–10].

Among the stress conditions faced by probiotic microorganisms during the gastrointestinal transit are the gastric environment and the intestinal residence. The adaptation mechanisms of probiotic bacteria to gastrointestinal stress are related to the innate or intrinsic and adaptive mechanisms identified in probiotic strains in response to acid stress and to bile and bile acid stress [11–13].

The aim of this study is to examine all the factors related to the development of stress throughout the production process of probiotics. The stress conditions that occur both in the stomach environment and in the residence of probiotic cells in the intestinal tract are also examined. Finally, in this study, attention is also focused on the improvement of multiple stress tolerance of lactic acid bacteria (LAB) as these are the microbial strains widely used as probiotics.

## Stress Conditions Faced by Probiotic Microorganisms During Food Manufacturing

Nowadays, microorganisms of probiotic properties belong to different classes of microorganisms including yeasts such as *Saccharomyces boulardii* and *Kluyveromyces lactis*, Gram-positive non-spore forming bacteria such as those belonging to former *Lactobacillus* sp. and *Bifidobacterium* sp., and spore formers such as *Bacillus* sp.

Recently, *Bacillus* (*B.*) sp. such as *B. coagulans* become very attractive candidates in probiotic industries [14]. This is based not only on the ease of cultivation in higher biomass culture but also on their high resistance to stress conditions; it is easy to keep the high cell and spore number during the whole production chain. It stands out as a property the high temperature resistance of these strains makes industry able to use the many choices of cost-effective methods in downstream processes especially of drying and formulation.

In fact, different types of probiotic yeasts are used nowadays as probiotics in both feed, food, nutraceuticals, and cosmeceutical industries [15]. Yeasts do not have the capacity to produce thermos-resistant intracellular spores. Therefore, they are more sensitive to environmental stresses compared to spore-forming bacteria.

Most of the commercial known formula of probiotic bacteria includes the facultative anaerobic bacteria belonging to *Lactobacillus* sp. or the strict anaerobic bacteria *Bifidobacteria* sp. A new classification of *Lactobacillus* has recently been proposed that includes 23 new genera. The two big groups are non-spore-forming and thus exhibit high sensitivity to environmental stress. Therefore, most of the research related to probiotic protection during the production process is associated with these two groups of bacteria and will therefore be the focus of this review.

### Bioreactor Cultivation of Probiotic Microorganisms

The highly diversified groups of probiotic microbes for sure also possess a wide range of nutritional and cultivation condition requirements. However, for all microbes used, the main target is to produce high biomass at the shortest period of time with minimal production cost. During the transfer of production process from small shake flask level to large scale, different parameters are changed. However, large-scale production facilitates the possible improvement of the cultivation process based on the possible control of pH, aeration, and agitation compared to shaking flask. However, large-scale production has also its own problems and most specifically temperature (as cell growth is exothermic reaction in case of aerobic microbes), controlling the oxygen supply to the cells in case of high cell density culture and suppressing foam production in some cultivation processes. However, controlling cultivation conditions and shifting from one cultivation strategy to another are easier in large-scale manufacturing compared to smaller scale.

In addition, it is also necessary to produce cells of some resistance to stresses they are exposed to during downstream processing, storage, and in vivo applications. These include pH, dryness, and osmotic and thermal stresses. Cultivation strategy during scaling up of the process can be designed to improve microbial resistance through both medium formulation and cultivation conditions. The selection and design of the resistance development strategy do not only depend on the type of microorganism but also based on the downstream approach used, product form, and storage conditions of the final product. Some possible strategies to implement during biomass scaling are detailed below.

#### Adaptation to pH Stress

Adaptation of low pH is one of the main targets during biomass production of probiotics as it improves acid resistance during probiotic in vivo application [16]. Adaptation to pH stress is usually started in small scale as sequential adaptation to low pH before large-scale production. In large-scale production, acid resistance can be achieved by using two-stage continuous fermentation which can enhance multistress of the probiotic strains or using immobilized cultivation system. A recent study also reported that exposure of *Bifidobacterium breve* cells to multistress conditions (high temperature, low pH, and oxidative stress) during cultivation at very low growth rate can induce high amount of stress proteins to improve multistress conditions during processing and application [17].

#### Adaptation to Dryness Stress and Osmotic Stresses

Adaptation of cells to dryness stress is usually carried out by successive adaptation process to increase cell viability during processing and storage in dry form especially for non-spore-forming probiotics. This is usually carried out in adaptation to multistress factors such as osmotic stress and pH. The preadaptation strategy to osmotic stress using successive adaptive growth at elevated salt (mainly sodium chloride) and sugar (mainly sucrose) concentrations before production is common for LAB strains such as *L. plantarum*, *L. acidophilus*, and *L. casei* [18].

#### Production of Microbial Metabolites to Reduce Environmental Stress

Probiotics have the capacity to produce a wide range of bioactive metabolites. Some of these metabolites can play a protectant role against environmental stress such as polysaccharides. For example, production of polysaccharides by LAB can act as natural protective agents to increase cell viability during downstream process. The productivity of polysaccharides by *Lactobacillus* strains can be controlled by medium composition and cultivation conditions as well [19]. It has been also reported that kefiran production by *L. kefiranofaciens* in submerged culture can be improved by manipulation of osmotic stress and addition of surfactants [20]. Moreover, addition of carbon dioxide during anerobic probiotic cultivation such as for *Bifidobacterium longum* not only enhances biomass production but also increases polysaccharide production, which increases cell resistance to stress [21].

### Stress Conditions During Drying Processes

In the manufacturing of living probiotic cells, many aspects of these cells are affected such as survival, viability, and growth [22]. The tolerance tests, concerning the selection and evaluation of the strains of probiotic cells, in view of their application, should be combined with those concerning their viability after drying and also with those concerning the formulation of the final probiotic product to be offered to the consumer [23].

Both high and low-temperature adaptation is equally important during the biomass production based on the industrial platform design. If downstream platform includes a freeze-drying process step, cell adaptation to cold stress is crucial. On the other hand, adaptation of cells to high-temperature stress is important if the downstream process includes spray drying process, which is cost-effective. It has been reported that cells’ exposure to cold stress increases cell viability between 2 log colony forming units (CFU/mL) and 5 log units for *Lactiplantibacillus* (*L.*) *plantarum* and *Lacticaseibacillus* (*L.*) *paracasei*, respectively [24]. On the other hand, exposure to sublethal temperature during exponential growth phase for short period range between 10 and 90 min can enhance cell viability in some *Lactobacillus* between 10 and 1000-fold depending on the type of strains and fermentation protocol [25, 26]. It has been also reported that high-temperature adaptation during cultivation is associated with the change in cell membrane fatty acid composition [27].

Dehydration techniques such as spray drying, freeze-drying, and vacuum drying are used to produce powdered probiotics containing high cell densities. The technological processing steps can be very tempting for probiotics and cause the limitation of their health benefits. The effectiveness of probiotics is assessed by having at least 10^7^ CFU of beneficial microbes per gram, by plate count enumeration, at the end of their shelf life as it is proposed by the International Dairy Federation (IDF). Probiotics before being included in a food are grown in large numbers by industry and then recovered, concentrated, stabilized, transported, and stored before their final use. Microbial cells are usually preserved and offered in the form of frozen cells, lyophilized cells, or dried cultures [8, 28] in many fermented foods and supplements [29].

Through freezing and thawing of highly concentrated cultures, it is possible to induce cell injury and subsequent loss of viability [30, 31]. Heat is the key contributor in spray drying, affecting a significant number of cell components, as it is the main stress factor [29]. In spray drying, the structure of the viable probiotic cells is affected, specifically the cell membrane and the envelope, causing a decrease in their viability [8] while their metabolic activity is also reduced [32], as a result of the extreme temperature stress [28]. The prevention or limitation of such damages can be achieved by the combination of other techniques, like microencapsulation [33]. Extreme temperatures also from freeze-drying (lyophilization), freezing, and thawing; dehydration from freezing, drying, and/or encapsulation; and osmotic stress upon drying or thawing might affect the probiotic cells in the step of their preparation [8]. Through drying, the cells are quiescent, metabolically inactive, and kept that way until consumption [34]. Dehydration stresses the cell membrane leading to cell death. When water is removed, there is an increase in the ratio between cell surface area and cell volume leading to membrane deformation [29]. During lyophilization, extracellular ice crystals form resulting in an increase in the concentration of medium solutes, causing osmotic stress [34, 35]. Although it is not entirely clear what happens to the inactivation mechanisms in spray drying, modifications in lipid membrane structure and protein denaturation are likely caused by high-temperature exposure, with ribosomal damage being the primary cause of cell death [29].

Fluidized bed drying also causes losses in cell viability due to osmotic stress, excessive dehydration, and oxidative stress [13]. In fluidized bed drying, the probiotic cells are minimally threatened at the typical temperatures used in this technique, up to a moisture level of 15%, while they are more threatened with a decrease in the water activity (*a*_w_) of the dry material [34].

Aryaee et al. [36] studied the survival of *L. plantarum* mixed with blend of fruit juice powder spray and freeze dried. Probiotic cells showed greater viability by freeze-drying compared to spray-drying, which may be attributed to harsher conditions in spray-drying compared to freeze-drying. High survivability in both freeze-dried and spray-dried powders was also demonstrated. The high survivability was also attributed to the use of protective agents and especially the use of malt extract [36]. Malt extracts were also used to increase the viability of *L. plantarum* in high acidic fruit beverages [37]. Similar results were obtained by Rishabh et al. [32] in which survivability of probiotic strains isolated from the Gundruk (a traditional Indian fermented vegetable food product) was studied among others. The lyophilized carrot juice probiotic powder showed higher viability than the spray-dried form as it had good storage stability reaching 1 month (6–7 log CFU/g).

### Food Matrix

The constituents in food, viz*.*, sugars, salts, aroma compounds, natural/artificial flavoring, and coloring agents being the main ingredients in food matrix, could be preservative, impartial, or harmful to probiotic stability [38]. Hereafter, the rapport of probiotics with these distinct food ingredients has a main role in their existence. Naturally, for fermented and non-fermented products, and throughout their storage, these elements could radically touch the probiotic viability and their growth [39, 40]. For instance, higher NaNO_2_ levels, frequently employed in meat system preservation, provoke a challenge to probiotics in fermentation [41]. On the other hand, several growth agents like glucose, minerals, vitamins, whey protein hydrolysates, casein, and yeast extract, added in dairy products, could enhance the growth rate of *Bifidobacteria* and *Lactobacilli* [42]. Practically, in the course of storage, these compounds possess helpful properties on the probiotic survival [43, 44]. Whey protein, hydrolysate acid of casein and tryptone, could stimulate the growth of the probiotic strains (*L. acidophilus* and *Bifidobacteria*) by allowing cell nutrition [45, 46]. This fact can be explained by the dropping of the redox potential of the medium and the elevation of the buffering capacity of the medium, resulting in pH decrease [47]. Alternatively, during milk fermentations steps, probiotic (*L. acidophilus* La-5 and *L. rhamnosus* Lr-35) growth was reduced; nonetheless, their survival was developed after storage [48]. In this vein, it was concluded that disaccharides could stabilize the cell membrane [49]. For instance, sorbitol can avoid the damage of the cellular membrane stabilizing protein functionality [50–52]. On the other hand, some prebiotics as well as oligosaccharides have an affirmative effect on *Bifidobacteria* viability in food products during storage [53, 54]. Elevated fat content, anaerobic conditions, and buffering potential of the cheese matrix could keep the probiotic cells in the final product throughout intestinal transition [55]. By virtue of the higher values, milk buffering capacity may result in advanced viability of probiotics in dairy fermented products [56, 57]. Additionally, food dry matter could absorb H ions, leading to the organic acid production [58]. Interestingly, delivery of viable probiotic *Lactobacilli* and *Enterococci* to the gastrointestinal tract exhibited an additional protecting consequence in cheddar cheese as a food carrier compared to yogurt [59].

An additional factor manipulating the stability of probiotic shelf-life stability was the moisture content of probiotic products [60]. In this line, bacterial survival was conducted by the storage in the existence of both O_2_ and moisture [61]. Water amounts disturb (i) the viability after drying and its rate of loss during subsequent storage [62]. The optimum moisture content for storage of freeze-dried *L. salivarius* subsp. *salivarius* ranged between 2.8 and 5.6%. Increasing the relative humidity (RH) of the environment at which the samples were stored caused an increase in water mobility and in the rate of loss in viability. It should be noted that *a*_w_ = 0.7 led to a decrease of a 10 log_10_ cycle of *L. rhamnosus* GG within 14 days of storage [63, 64].

The packaging features could impact on the probiotic viability. Generally, thickness of the packaging materials, gas, light permeability of polymer, and techniques like active/intelligent packaging systems may have an impact on probiotic viability [57]. In addition, parameters as *T* and relative humidity could touch the gas permeability of the packaging material and thus disturb the probiotic viability [65]. The incorporation of *Bifidobacterium longum* NCTC11818 in buffalo curd could establish a probiotic product, and the probiotic strain could survive 3 days in clay pots at 29 °C [49]. Additionally, freezing reduces post-fermentation acidification and extends the strain viability. Interestingly, compared to non-inoculated curd, probiotic buffalo had higher sensory scores. Cruz et al. [66] assessed the probiotic stability in yogurts fortified with glucose oxidase and enveloped in diverse plastic systems with different O_2_ permeability transfer rates ranging from 0.09 to 0.75 mL O_2_/day. At lower O_2_ permeability rates, tested polymer exposed a higher concentration of the probiotic bacteria in yogurts during refrigerated storage. Moreover, inoculated samples displayed a higher extent of post-acidification and organic acid production, since awfully low O_2_ permeability of glass packages contributed to survival of probiotic cultures.

### Storage

After probiotic incorporation on food matrix, certain processes were needed for the stabilization, protection, and product maintenance until being consumed.

#### Heat Stress

Heat handling, frequently achieved throughout food manufacturing, requires high *T* that are unfavorable to the majority of microorganisms. Consequently, after subsequent processing, it is desirable to include proper probiotic(s) [67]. At elevated temperatures, between 45 and 80 °C, some probiotic LAB can live for hours. For instance, *L. fermentum* KGPMF28 and KGPMF2 *L. fermentum* KGPMF28 are able to grow at 45 °C/24 h [68]. At 55 °C–15 min (heat shock) and 45 °C–30 min (thermal adaptation), *L. plantarum* strains (Lp 813 and Lp 998) displayed comparable behavior against thermal, osmotic, and oxidative stress factors [69]. Selected thermal adaptation improved the thermal resistance of both strains by 2 log orders. The relevance of cell technological resistance was demonstrated by these authors when choosing possible “probiotic” cultures. Similarly, Haddaji et al. (2015) [70] found that *L. casei* cells persisted cultivation at 65 °C, which proves that bacteria are talented to withstand such adverse environments.

Physiologically, high *T* could develop the membrane fluidity and, consequently, interrupt the cell activity [71]. To prevent the degradation and denaturation of probiotic LAB, Chen et al. (2017) [72] confirmed that *L. kefiranofaciens* M1 possesses a diversity of adaptation mechanisms, comprising the production increase on specific evolutionarily conserved proteins. These proteins include HSPs and enzymes, viz*.*, PtsI, DnaK ProS, GroEL, and GroES, that take a leading role in endorsing proper folding and a consequent translocation of nascent polypeptides [73]. In addition, probiotics that grow under heat stress possess saturated and straight-chain fatty acids, which offer the correct fluidity required for membrane function [74]. The expression of DNA-binding proteins stands as an alternative approach to biomolecules keep akin to DNA [75]. The efficiency of these preserved proteins in cells from heat stress can differ from species to species. For example, a heat tolerance study within the *Lactobacillus* genus displayed that genetic variation/environmental factors, such as culture media, NaCl concentration, *a*_w_, and pH, expressively could impact on strain resistance to heat stress [76].

#### Cold Stress

Some probiotics could grow at *T* < 15 °C and adapt in numerous cold environments. In this line, the probiotic viability during cold temperature storage of fermented food before consumption is a decisive factor for their functional characteristics [77]. A rapid temperature decrease can convince physiological stresses via reducing membrane fluidity and DNA altering supercoiling and RNA, which may disturb replication, transcription, and protein production [78]. In cold *T*, the components and enzymes of probiotics developed a rigid, provoking toxicity [79]. Additionally, at very low temperatures, ice crystals formed probiotic cell membranes, injuring or killing the cells [80]. Furthermore, cold stress conditions should also kill cells after freezing. As an example, *B. subtilis* growing is detained upon a *T* deceleration, causing the hindering probiotic protein synthesis [81]. LAB probiotics could overcome these damaging impacts and be functional at low *T* by generating antifreeze and CSPs, by controlling the expression of cold-induced genes (as antiterminators), which improve detrimental effects related to cold environments [82]. On the other hand, probiotic LABs are familiar to produce cold-adapted enzymes that sustain activity at freezing *T*° and provision both transcription/translation [83]. Some probiotic strains are also qualified by antifreeze proteins joined to ice crystals and stop them from intense cells [84].

## Stress Conditions Faced by Probiotic Microorganisms During Gastrointestinal Passage

**Fig. 1:**
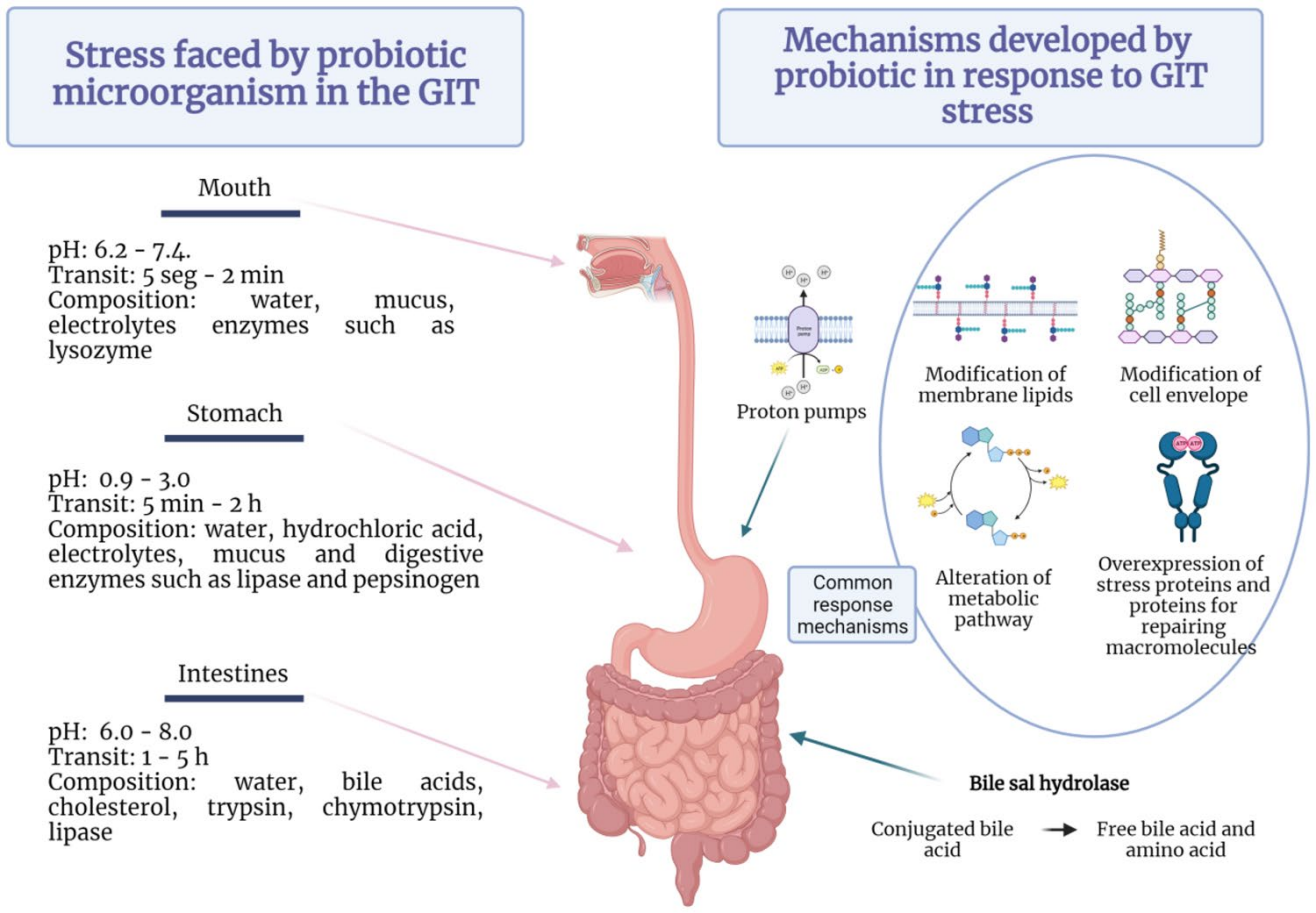
The passage of probiotics through the GIT. Stresses coped with and coping mechanism developed. Figure adapted from images created with BioRender.com

After ingestion, the survival of probiotics is greatly compromised by the unique environment of the human gastrointestinal tract (GIT), where they are exposed to a plethora of harsh physicochemical conditions [85]. Salivary enzymes, the low pH of the stomach, and the presence of bile in the intestine are crucial barriers that probiotic bacteria have to overcome to exert their beneficial action (Fig. 1).

Orally administered probiotics are initially exposed to saliva, a complex fluid containing water, mucus, electrolytes, and enzymes, with a pH in the range between 6.2 and 7.4, among which lysozyme stands out for its antimicrobial properties [86, 87]. In addition, saliva contains immunological components and receptor proteins that can block microbial surface adhesins and modulate colonization in oral tissues. However, available studies suggest the zero impact of saliva components on the viability and colonization capacity of probiotics, as the exposure time is transient [88].

### Stomach Environment

The next critical stage in the probiotics’ long journey through the GIT is the stomach, where the most detrimental condition is created by acidic gastric fluid (Fig. 1). Between 1.5 and 2.0 L of gastric juice is produced per day, consisting of water, hydrochloric acid, electrolytes, mucus, and digestive enzymes such as lipase and pepsinogen, among others. During digestion, the secretions of the parietal cells mix with the food bolus so that the pH of the stomach oscillates between 0.9 and 3.0, during a transit time of 5 min to 2 h [11]. An antimicrobial effect is exerted by the unique composition and low pH of gastric secretions, in which allochthonous microorganisms, such as probiotics, can be rapidly destroyed.

An acidic environment can cause a reduction in bacterial intracellular pH (pHi), alter the anion pool, affect the integrity of DNA bases, and cause denaturation of essential enzymes, including a decrease in the activity of glycolytic enzymes and F1F0-ATPase proton pumps [85, 88].

As it is known in bacteria, F_1_F_0_-ATPase generates ATP when extracellular protons cross the cell membrane into the cytoplasm across a pH gradient [89]. However, in an acidic environment, the accumulation of H^+^ causes the pumps to consume ATP, depleting the available energy and leading to cell death [85].

In addition, low pH can damage the composition, integrity, and functionality of plasma membranes [85, 90]. Indeed, exposure to acid can induce several modifications in cell membrane composition of proteins and lipids, alter the diffusion of molecules, and modify peptidoglycan components. Considering that cell membranes provide a constant intracellular environment to maintain cellular activities and that they also participate in the stress response of cells, these modifications could lead inexorably to death in non-adapted cells [85]. For example, potential probiotic *L. plantarum* UBLP40 survival was significantly reduced (*p* ≤ 0.0001) to zero within 1 h at pH 1.0 compared to the control at pH 7.3, while at pH 2.0 and 3.0, 73–95% and 93–100% survival was detected, respectively, depending on exposure time [91]. Furthermore, Sesín et al. (2023) [92] reported that 15 out of 17 LAB isolated from goat cheese showed a survival rate higher than 75% after 3 h of exposure at pH 2.5. Finally, when different strains of *Lactococcus lactis* and *Leuconostoc lactis* were incubated for 12 h in MRS at pH 2.0, 3.0, and 4.0, a viability of 24–27, 8–9%, and 6–7%, respectively, was recorded. On the contrary, exposure of cells to simulated gastric juice at pH 3.0 for 3 h showed survival of 100% [93].

### Intestinal Stay

Once in the gut, the main factors that regulate the establishment and permanent existence of bacteria include the disruptive activity of digestive enzymes, sudden pH changes, detergent properties of bile acids (BAs), and molecules produced by the host’s immune system [94, 95]. Indeed, when passing from the stomach to the intestine, probiotics are first confronted with a sharp rise in pH from 2.0 to 6.0 mainly due to the presence of bicarbonate (Fig. 1). This abrupt change can damage membranes and alter the structure of proteins and DNA, among others, thus compromising microbial viability [11]. In addition, the cells are exposed to the action of pancreatic secretions, mainly composed of digestive enzymes such as proteases (trypsin and chymotrypsin), amylases, and lipases [96]. The digestive enzymes have been shown to reduce the adhesion to intestinal mucus of probiotic strains [97]; however, the effect of pancreatic secretions on microbial viability has not been studied in depth.

Bile secretions are produced and secreted by the liver, stored in the gallbladder, and secreted into the duodenum. It consists of an alkaline solution with a pH between 7 and 8 whose main components are BAs, biological detergents involved in the emulsification and absorption of dietary lipids. The concentration of BAs ranges from 12 mM in the duodenum after a high lipid intake to 2 mM in the ileum due to active reabsorption [98]. The primary BAs, chenodeoxycholic acid and cholic acid, are synthesized in the liver from cholesterol, in a multienzyme process [94]. Before leaving the liver, its steroid nucleus is conjugated with taurine or glycine (predominantly in humans) to increase its solubility. Conjugated BAs are accumulated in the gallbladder and released to the duodenum after food intake, contributing to the solubilization of ingested lipids. Then, they are reabsorbed and returned to the liver via enterohepatic circulation process [99].

Non-reabsorbed BAs are strongly modified by intestinal microbes and primary BAs are converted to over 20 different secondary metabolites. Indeed, composition of BAs is strongly affected by gut bacteria, and reciprocally, they are the major regulators of shape and composition of gut microbiota [100]. The main reaction of BA metabolism is the hydrolysis of the amide bond to release the free bile acid plus the amino acid, reaction performed by microbial enzymes collectively called bile salt hydrolase (BSH) [96].

Bacterial membranes are the main target of the antimicrobial action of BAs due to their amphipathic character that confers detersive properties. In general, LAB are thought to accumulate in the lipid bilayer and then penetrate into the cell interior. In this sense, antimicrobial activity is related to the chemical structure and hydrophobic properties of BAs as they can diffuse and accumulate more easily in the lipid bilayer [101].

Electron microscopy studies revealed that exposure to BAs resulted in alterations in the membranes which showed folding and budding [102], while the cytoplasm decreased and the membranes became thin and rough [103]. Moreover, BA incorporation into the phospholipid bilayer can interfere with phospholipid molecules’ normal arrangement thus leading to a loss of membrane integrity in many probiotic bacteria.

In bacterial cells, the proton driving force, composed by the transmembrane electrical potential (ΔΨ) and the pH gradient (ΔpH), supplies the electrochemical energy needed for cell growth and ATP synthesis, pH homeostasis, membrane transport, motility, stress resistance, cell division, electrical communication, and environmental sensing [104]. Consequently, disruption of the proton motive force has severe consequences for the cell, even leading to cell death. Dissipation of ΔΨ and ΔpH accompanied by intracellular acidification and intracellular ATP depletion and leakage of essential ions and small molecules was reported in probiotic strains exposed to conjugated and free BAs [98, 105–107]. Moreover, oxidative damage to DNA and RNA and protein misfolding could be induced by BAs [108, 109]. In this regard, Bustos et al. [109] reported that deoxycholic acid induces greater changes in the secondary structure of a model protein than taurodeoxycholic acid, modifying surface charges and inducing the formation of protein aggregates, resulting in a loss of activity. In addition, deoxycholic acid interacts in the active site of the enzyme while taurodeoxycholic acid does not, probably due to the presence of taurine as shown by molecular docking studies.

### Understanding the Adaptive Mechanisms of Probiotic Bacteria to Gastrointestinal Stress

Microbial responses to stress, including those of the GIT, are multifactorial phenomena; however, they can be broadly classified into (i) innate or intrinsic mechanisms and (ii) adaptive mechanisms. The former includes structures and metabolic pathways naturally present in the microbial cell that allow tolerance to the stressor. Adaptive responses involve genotypic and phenotypic modifications, which may be associated with mutations that arise following the exposure of cells to stress and allow microorganisms to survive in its presence [11]. Probiotic microorganisms have developed common coping strategies to deal with both acid and bile stress, as well as other types of environmental stress, as mentioned above. The most common defense mechanisms include regulation of energy production by modulation of different metabolic pathways, modification of cell envelope and membrane lipid composition, overexpression of chaperones, stress proteins, and macromolecule repair enzymes, among others (Fig. 1, Table 1).

**Table 1:** Acid and bile stress response in various probiotic bacteria

Response mechanism	Stress	Strains	Reference
Innate mechanisms			
pH homeostasis	Acid Bile Bile	*Lactobacillus acidophilus* Several lactobacilli *Limosilactobacillus reuteri*	[110] [98] [107]
Regulation of membrane fluidity	Acid	*Lactobacillus casei*	[111]
Efflux pumps	Bile	*Limosilactobacillus reuteri*	[106]
Architecture and composition of the cell membrane	Bile	*Bifidobacteria* *Limosilactobacillus reuteri* *Lactobacillus gasseri* *Lacticaseibacillus paracasei*	[112] [102] [113] [114]
Adaptative mechanisms			
Stress responsive proteins such as small heat shock proteins	Bile	*Limosilactobacillus reuteri* *Limosilactobacillus fermentum* *Lactobacillus salivarius* *Lactobacillus johnsonii* *Lactobacillus mucosae*	[115] [116] [117] [118] [119]
Accumulation of ammonia by overexpression of genes encoding ammonium transporters and cystathionine gamma-synthetase was upregulated	Acid	*Bifidobacterium longum*	[120] [121]
Expression of proteins involved in peptidoglycan synthesis	Acid Bile	*Bifidobacterium longum* *Lactobacillus fermentum* *Pediococcus pentosaceus*	[122] [120] [116] [123]
Expression of transport and deamination of branched-chain amino acid proteins	Acid	*Bifidobacterium longum* *Lactiplantibacillus plantarum*	[121, 122] [124]
Expression of F_0_F_1_-ATP synthase	Acid Acid Acid	*Lacticaseibacillus rhamnosus* *Bifidobacterium longum* *Lactobacillus casei*	[125] [121] [126]
Expression of bile export systems and efflux pumps	Bile	*Bifidobacterium breve* *Akkermansia muciniphila* *Limosilactobacillus reuteri* *Lactobacillus salivarius* *Lactobacillus mucosae*	[127] [128] [129] [117] [118]
Maltose utilization pathway	Bile	*Lactobacillus salivarius* *Bifidobacterium animalis*	[117] [130]
Expression of bile salt hydrolase	Bile	*Bifidobacterium animalis* *Bifidobacterium longum*	[131] [121] [132]

Resistance strategies developed by probiotic bacteria to GIT stresses can be studied using physiological, biochemical, and genetic analyses, while omics approaches have recently proved valuable in discovering new biomarkers of stress resistance. Indeed, the advent of omics technologies has enabled a deeper and more holistic understanding of probiotic biology and response mechanisms, which has prompted the search for new ways to screen for the best candidates.

### Innate and Adaptive Mechanisms Identified in Probiotic Strains in Response to Acid Stress

Since stomach acidity represents one of the main survival challenges for probiotic microbes, sophisticated mechanisms at the physiological and molecular level have been devised to survive and adapt to acid stress. In addition, the mechanisms of acid tolerance in different probiotic microorganisms have been investigated by many different approaches. Some of the responses to acid stress in various beneficial bacteria are listed in Table 1.

Maintaining pH homeostasis, *i.e.*, pH regulation inside and outside the cell, is a strategy that some microorganisms have developed to resist acid stress [133, 134]. As a strategy, certain yeasts and bacteria, when exposed to a changing extracellular pH, can maintain a near-neutral pHi, quite stable, and generate unfixed proton gradients [85]. However, maintaining a stable pHi requires significant energy consumption, with the immediate result the restriction of the growth and metabolism of microbes. In contrast, most acid-tolerant microbes, such as LAB, have developed a different strategy for survival, by maintaining a constant pH gradient rather than a constant pHin [89]. Thus, the pHi of these microbes decreases as the pHext decreases but remains at a level above the pHex [135]. Using this strategy, it is advantageous for LAB because proton translocation consumes energy, and these fermenting bacteria obtain significantly less energy from sugar metabolism than aerobic bacteria. A positive ∆pH is critical for many cellular bacterial processes, such as cell growth, energy uptake by ATP synthesis, DNA replication, transcription, and translation, among others. However, as acid continues to enter the cell, a critical concentration is reached after which the pHi decreases sharply; the ∆pH collapses or approaches zero, disrupting vital cellular processes and resulting in a loss of cell viability [98]. Therefore, pH homeostasis maintenance is required for survival of microbes in acidic environments.

In this sense, microbial cells can maintain pH homeostasis by different strategies, including (i) modulating cell membrane permeability and modifying channel size to restrict proton entry, (ii) removing excess protons from the cytoplasm via the proton pump, (iii) diverting proton entry by formation of chemiosmotic gradients via potassium ATPases, and (iv) maintaining membrane fluidity, characteristics that are determined by the acyl fatty acid chain composition and the head group [85]. In a study of wild-type *L. casei* Zhang and its acid-resistant mutant, it was observed that in response to acid stress, the cell membrane fluidity decreased and reduction of acid damage was achieved by a change in membrane’s fatty acid composition. Compared to the wild-type strain, the mutant had higher proportions of unsaturated fatty acids and a longer average chain length [111].

Using omics tools, key mechanisms of adaptation to acid stress were identified in probiotic strains. In a comprehensive work by Wei et al. [120], the differential gene expression of the probiotic strain *Bifidobacterium longum* JDM301 and its stress-adapted derivative strain was evaluated. The resistant strain was obtained by 150 batch subcultures. Both strains were grown at an initial pH of 6.5 or pH 3.5, and high-throughput RNA sequencing was carried out to analyze changes in the gene expression profile. Notably, genes encoding ammonium transporters and the enzyme cystathionine gamma-synthetase were upregulated under acid stress in both strains. Cystathionine gamma-synthetase is responsible for the synthesis of cystathionine, which can then be converted to ammonium. Similar results were reported by Sánchez et al. [121] in an acid-resistant mutant of *Bifidobacterium longum*, demonstrating the importance of ammonium in the neutralization of excess protons. In addition, some genes involved in peptidoglycan synthesis and fatty acid metabolism were upregulated in *Bifidobacterium longum* JDM301 under both normal and acid stress conditions [120]. These findings suggest that cell envelope and membrane repair are important in both adaptation and acid resistance of the strain since they are the first targets of action of several stresses. Similarly, Jin et al. [122] reported overexpression of proteins involved in peptidoglycan synthesis, accompanied by an increase in their production, in *Bifidobacterium longum* BBMN68 pre-stressed by exposure to a sublethal pH and subsequently subjected to pH 3.5. In addition, pre-stressing increased the abundance of proteins involved in energy production, amino acid metabolism, and ATP and NH_3_ content, thiols, and H^+^-ATPase activity relative to uninduced cells.

Other proteins, normally affected in acid-exposed probiotic cells, belong to the following functional categories: translation and transcription, macromolecule protection and repair, stress proteins such as chaperones, energy production and conversion, carbohydrate transport and metabolism, and amino acid transport and metabolism, in particular, the transport and deamination of branched-chain amino acids, which has been postulated as a mechanism for maintaining bacterial internal pH [120, 122, 124]. Surprisingly, repression of genes involved in cell division, in particular the tubulin analogue FtsZ, was observed, a finding that has also been described in bacteria under other types of environmental stress [120].

### Innate and Adaptive Mechanisms Identified in Probiotic Strains in Response to Bile and Bile Acid Stress

Given the complicated nature of bile stress, different defense mechanisms should be deployed by microorganisms in order to overcome its presence. BAs are the major constituents of bile and are mainly responsible for its antimicrobial effect. Moreover, tolerance to BAs is strain-dependent and shows extreme variability even within the same genus or species. As already mentioned, omics technologies allowed the characterization of the responses of probiotic bacteria to different types of stress, including the presence of bile and bile acids. Furthermore, using omics approaches, specific bacterial biomarkers were proposed to find the best strains with probiotic potential. Some of the responses to bile stress in various beneficial bacteria are listed in Table 1.

Resistance of the presence of bile in probiotic bacteria by the intrinsic resistance mechanisms is related to changes in the architecture and composition of the cell membrane and the presence of efflux pumps and by the ability of the cells to maintain intracellular homeostasis. In addition, BSH enzyme has been recommended to participate in a potential detoxification mechanism for the resistance of the presence of BAs [94, 113, 136]. Accordingly, omics studies show that the main genes and proteins expressed in response to bile in probiotic bacteria are related to fatty acid biosynthesis, the metabolism of carbohydrates, amino acids and nitrogenous bases, bile salt transporters, and stress response proteins [113, 115, 117, 123, 128].

Exposure to bile and bile acids has been reported to induce changes in membrane lipid and fatty acid composition in probiotic Bifidobacteria and Lactobacilli, which may contribute to their stress tolerance [112, 137]. To evaluate the adaptation mechanisms of a probiotic strain of *L. gasseri* JCM1131T, a BA-resistant strain was obtained by Kato et al. [113] through pre-exposure to sublethal concentrations of cholic acid. Adaptation resulted in reduced cell membrane damage and increased abundance of long-chain sugar glycolipids, as well as a doubling of cardiolipin content. In addition, cardiolipin reduced phospholipid vesicle solubilization after cholic acid exposure, suggesting that it plays a significant role in bile acid resistance and cell membrane maintenance. Similar finding were recently reported by Shimizu et al. [113] in *L. paracasei* strain Shirota. In this line, it was reported that the addition of soy lecithin, as a source of phospholipids, is able to modulate the surface properties and bile resistance of *L. plantarum* strains, confirming the important role of membrane composition in bacterial tolerance to bile [138]. In addition, a recent study showed that the addition of a squalene synthase inhibitor increases the susceptibility of *Akkermansia muciniphila* to BAs by changes in membrane structure. *Akkermansia muciniphila* is an intestinal commensal bacterium that has recently attracted the attention of researchers due to its probiotic effects related to amelioration of obesity and metabolic disorders and modulation of the host immune response [128]. In addition, transcriptome analysis of *Akkermansia muciniphila* DSM 22959 showed the upregulation of proteins associated with hopanoid production in the presence of BAs. These results indicate a probable BA tolerance in *Akkermansia* by hopanoid production, associated with membrane permeability [128].

On the other hand, the cell wall is also crucial for the maintenance of cell homeostasis under conditions of environmental stress. Peptidoglycan synthesis is a complex phenomenon, controlled by numerous proteins encoded by the bacterial genome. In this regard, Ali et al. [116] detected that 35 proteins related to cell wall biosynthesis were upregulated in *Lactobacillus fermentum* NCDC 605 in response to bile. Furthermore, research showed that cell wall synthesizing enzyme in Gram-positive and Gram-negative bacteria, UDP-N-acetylmuramate-L-alanine ligase, was upregulated and overexpressed in *P pentosaceus* M41. This enzyme is related to the strain’s tolerance to different types of stresses, such as acid and thermal as well as BAs [123]. Finally, enzymes involved in cell surface charge modification and a hemolysin-like cell envelope protein were overproduced in the presence of bile in *Lactobacillus salivarius* Ren, probably to prevent bile adsorption [117].

The upregulation of efflux transporters was reported in many probiotic strains to reduce the intracellular accumulation of BAs [117]. In this sense, transcriptome and proteomic analysis of *Akkermansia muciniphila*, *L. reuteri*, *L. salivarius*, and *Lactobacillus mucosae* showed that transporter gene clusters were upregulated in the presence of bile and BAs [117, 118, 128, 129].

In response to stress, bacterial cells increase carbohydrate metabolism because maintaining fundamental cellular processes under these conditions requires high energy production. In this regard, Ali et al. [116] identified 53 proteins involved in various carbohydrate metabolic pathways, including glycolysis, pentose phosphate pathway, galactose synthesis and glutamine pathways, while exoproteomes analysis of *L. johnsonii* and *L mucosae* revealed overexpression of proteins involved in glycolysis, such as L-lactate dehydrogenase, fructose-bisphosphate aldolase, glucose-6-phosphate isomerase, triose phosphate isomerase, phosphoglycerate kinase, phosphoglycerate kinase, and phosphofructokinase [118, 119]. These pathways aim to provide ATP to meet the energy demands of cells under stress conditions. Moreover, Wang et al. [117] employed transcriptomic and proteomic analysis to describe adaptation mechanism of *L. salivarius* Ren exposed to 0.75 g/L ox-bile. Surprisingly, the maltose utilization pathway, whose metabolism produces more than twice as much ATP as glucose metabolism, was overexpressed in the presence of bile. It was previously reported on the preference of maltose over glucose compared to the parental strain for a bile-resistant derivative of *Bifidobacterium animalis*, probably due to non-availability of glucose in the distal colon [130].

Proteins and genes involved in transcription and translation, as well as in the general stress response, are increased in the presence of bile, as this stress affects several cellular systems. These include a large number of highly conserved proteins in bacteria that are often involved in the maturation of new proteins, refolding or degradation of denatured proteins, and DNA repair [139]. The expression of these stress-response proteins was also induced by other stresses including acid, heat, cold, and osmotic stress, suggesting that this system is a general adaptive mechanism in probiotic bacteria [90, 139]. Chaperones such as several heat shock proteins (Hsp), GroEL, GroES, DnaJ, and DnaK as well as elongation factor were induced to counteract with bile and BAs in *L. reuteri* CRL 1098 [115], *L. fermentum* NCDC 605 [116], *L. salivarius* Ren [117], *L. johnsonii* PF01 [118], *L. mucosae* LM1 [119], among others.

Finally, some evidence suggests that BSH activity might affect bile tolerance in some Gram-positive bacteria as part of a cell detoxification strategy [136, 140]. As stated above, the main reaction in BA metabolism is performed by BSH enzymes. BSH activity has been described in bacterial genera of intestinal origin, including former genus *Lactobacillus*, *Bifidobacterium*, *Clostridium*, *Enterococcu*s, *Listeria monocytogenes*, and *Brucella abortus*, among others but not exclusively. The potential role of BSH activity in detoxification has been discussed, as free BA are less soluble, precipitate at intestinal pH, and leave the GIT with the feces, decreasing their interaction with gut bacteria [136]. An early metagenomic study [141] links resistance to conjugated BAs of intestinal microorganisms with the presence of BSH activity. An interesting work by O’Flaherty et al. [142] investigated the presence of *bsh* genes in 170 lactobacillus genomes. The results revealed that species harboring *bsh* genes are mainly associated with vertebrate-adapted niches, suggesting a selective pressure on lactobacilli to evolve and adapt to specific environments.

Changes in BSH expression at the transcriptomic and proteomic level in response to bile have been reported in previous studies. A proteomic study revealed the repression of the expression of the BSH enzyme by a bile-resistant strain of *L. plantarum*, while sensitive strains showed no change [143]. Moreover, a bile-adapted strain of *Bifidobacterium animalis* showed overexpression of the BSH enzyme compared to its wild-type counterpart. The same thing was observed in *Bifidobacterium longum* exposed to an intestinal environment [121, 132, 133]. Finally, neither *Enterococcus faecalis* V583 nor *L. reuteri* ATCC 23272 modified BSH enzyme expression at proteomic level in the presence of bile [144, 145].

## Improvement of Multiple Stress Tolerance of Lactic Acid Bacteria

The challenge of improving probiotics survival rate during the different steps of the bioprocess that includes biomass production, dehydration (freezing, freeze-drying, spray drying), and long-term storage of the products is included in probiotic production. A probiotic must be able to tolerate exposure to a number of different stressors during its lifetime. The stress tolerance of *Lactobacillus* species can be strain-specific, so stress tolerance is a crucial factor in selecting strains for use as probiotics or in other industrial applications. Researchers and manufacturers often assess these properties when characterizing and selecting specific Lactobacillus strains for various purposes. By reason of the various stressors that can affect the viability of probiotics during production and storage was require a number of technological strategies to help minimize the effect of stressors.

### Strategies in the Probiotic Bioreactor Production Stage

The physiological state of the pre-process culture is key to the intrinsic tolerance of the cells to stress conditions [146–148]. Bacterial cells can be prepared to cope with different adverse environments by modifications in fermentation conditions. These include culture medium composition, temperature, pH, culture age, and atmosphere (gas injection). Lactic acid is produced by LAB due to their type of metabolism, leading to a decrease in the pH of the culture. Although LAB can grow in a wide pH range (3.5–6.5), the cultures in stationary phase are subjected to acid stress which limits their growth and may affect their resistance to subsequent stress factors. Thus, avoidance of cell injury and improvement of biomass yield are achieved by pH-controlled fermentations [149]. Contrary, Ai et al. [150] reported on *L. bulgaricus* Q7 where the average growth rate and the final biomass were higher under free pH conditions. At this point, it is necessary to differentiate biomass yield with the possibility of generating a technologically robust biomass with a resistance to multiple stress situations. Inevitably, the biomass obtained in bioreactors must subsequently withstand multiple technological processes and the metabolic condition of the biomass will determine its survival. Recently, the effect of culture parameters (pH, growth phase) on cell viability and heat tolerance of probiotic *L. rhamnosus* CRL1505 was evaluated [149]. These research works reported that running fermentations at pH 5.5 and harvesting the cells at the exponential phase are the best conditions for obtaining a high live biomass yield capable of overcoming heat stress.

The production of probiotic cultures on an industrial level only considers the highest biomass without taking into account their condition. Thus, they are generally harvested in the stationary phase, in which there is a high percentage of damaged cells, often unable to survive subsequent stress factors. If we consider the strategy of pH-controlled cultivation to minimize this damage, the literature search indicates that the results are strain-dependent. At pH 6.0, some probiotic cultures showed a low survival to heat, oxidative, and osmotic stress [149, 151].

Depending on the composition of the culture medium, during growth, some microorganisms accumulate compatible solutes to maintain osmotic equilibrium with the extracellular environment. Compatible solutes, including betaine, carnitine, and proline, refer to the accumulation of protective compounds. LAB do not synthesize compatible solutes and therefore depend on the environment to take them up [152]. Compatible solutes can facilitate the stabilization of proteins by microbial cultures and the cell membrane during osmotic stress conditions caused by low water activity during the drying process [153, 154]. In this regard, Huang et al. [148] proposed that cytoplasmic accumulation of inorganic polyphosphate (polyP) in *Propionibacterium freudenreichii* is osmotically induced and utilized as energy storage molecules and compatible solutes. The accumulation of polyP in this microorganism led to increased multistress resistance and, particularly, to increased survival after spray drying [153, 155]. Extensive studies of the synthesis of polyP in microorganisms have been reported due to its use in bacterial physiology. The synthesis of polyP by some probiotics depends on the concentration of phosphate in the culture medium and the growth phase, so the formulation of a suitable medium and the harvesting of biomass at the appropriate stage may be an appropriate strategy to favor the survival of the probiotic to the drying processes.

The synthesis of chaperone proteins is one of the protective strategies employed by probiotics to cope with heat stress [156]. Stabilization of protein structure and function can be achieved by this mechanism, thus contributing to maintenance of optimal metabolic performance under various stress conditions [157]. At this point, mutants with an overexpression of chaperones can be generated. Corcoran et al. [158] reported an overproduction of GroESL by the probiotic strain *Lactobacillus paracasei*. This strain showed (higher survival to spray and freeze-drying) compared to the unmodified wild-type strain.

Bacteria have evolved mechanisms (uptake and synthesis systems) for the accumulation of compatible solutes [157]. Three distinct uptake systems (BetL, Gbu, and OpuC) and a compatible solute synthesis system (ProBA) of the food pathogen *Listeria monocytogenes* have been discussed in probiotics [159]. The transcriptional control of the nisin-inducible promoter PnisA to assess the role of BetL (and thus betaine accumulation) in contributing to the survival of *Lactobacillus salivarius* UCC118 in a variety of stress situations might be responsible, according to some authors, for the betL gene (encoding the BetL betaine uptake system) [160, 161]. *L. salivarius* showed a significant increase in betaine accumulation and increased resistance to stress factors compared to the wild type.

Oxygen is an important factor for probiotic bacteria, as it affects both positively and negatively their growth. Most of the growth of probiotic lactobacillus species depends on the presence of electron acceptors as they are considered oxygen-tolerant anaerobes because they do not have a complete electron transport chain [162]. The presence of oxygen incorporated into the bioreactor by agitation can induce the production of reactive oxygen species (ROS) that lead to damage of cellular components, such as proteins, lipids, and nucleic acids [163]. Thus, one strategy is to add electron-accepting molecules [164, 165] to the substrate for aerobic culture and minimize agitation during production. By keeping oxygen at a sufficient minimum level, maximization of biomass yield and avoidance of oxidative stress can be achieved [164, 166]. In the most recent study of Rao et al., a significantly higher survival rate of *Limosilactobacillus* (*L.*) *reuteri* DSM 17938 was observed in the lyophilized product for cells cultured both in the presence and absence of oxygen (61.8% ± 2.4% vs. 11.5% ± 4.3) [12]. On the contrary, during drying, oxygen-sensitive bacteria, e.g., *Bifidobacteria*, due to the lack of oxygen in the drying environment, may result in a reduction in oxidative stress [13].

### Improvement of Survival During the Dehydration Process

Freeze-drying is an efficient and widely used technique for preserving probiotics. By offering increased stability and viability, freeze-drying allows probiotics to maintain their biological activity and health benefits during storage. Freezing and osmotic stress reduce bacterial enzyme activity [167, 168]. The freezing stage prior to water sublimation induces crystal formation, which stiffens LAB cell membranes and reduces membrane fluidity [92]. To overcome the adverse situation, LAB have implemented a number of adaptive mechanisms. Derzelle et al. [169] suggested that cold stress is alleviated by upregulation of cold shock proteins (CSPs) (CspL, CspP, and CspC) [170]. In addition, it is possible to increase cryotolerance by accumulation of compatible solutes. Overexpression of BteL, the betaine uptake system in *L. salivarius*, led to the accumulation of betaine glycine, which increased resistance during freeze-drying [161]. Survival of *L. bulgaricus* in freeze-drying was improved if the cells were subjected to osmotic stress [171]. Osmotic stress led to stress adaptation by overexpression of the compatible solute uptake system, leading to its accumulation and thus enhancing freeze-drying tolerance. Osmotic stress adaptation can be induced by the addition of salt and compatible solutes such as trehalose. Trehalose can improve the survival of LAB during freeze-drying [172]. The accumulation of intracellular trehalose caused by osmotic stress leads to improvement of survival during freeze-drying.

In addition, improvement of survival rate of LAB can be implemented by the exogenous addition of suitable protective agents and by adjustment of the freeze-drying process [173]. The literature suggests that the best approach to increase survival is the use of a cryoprotectant [172]. Cryoprotectants such as sucrose, lactose, sorbitol, and skim milk have been proven to be effective in improving the survival of probiotic bacteria during freeze-drying and storage [174]. The synergistic combination of cryoprotectants, namely, 6% sucrose/8% skimmed milk/4% monosodium glutamate, gave a high survival rate of *Streptococcus thermophilus* by lyophilization of 90.59% [175].

**Fig. 2:**
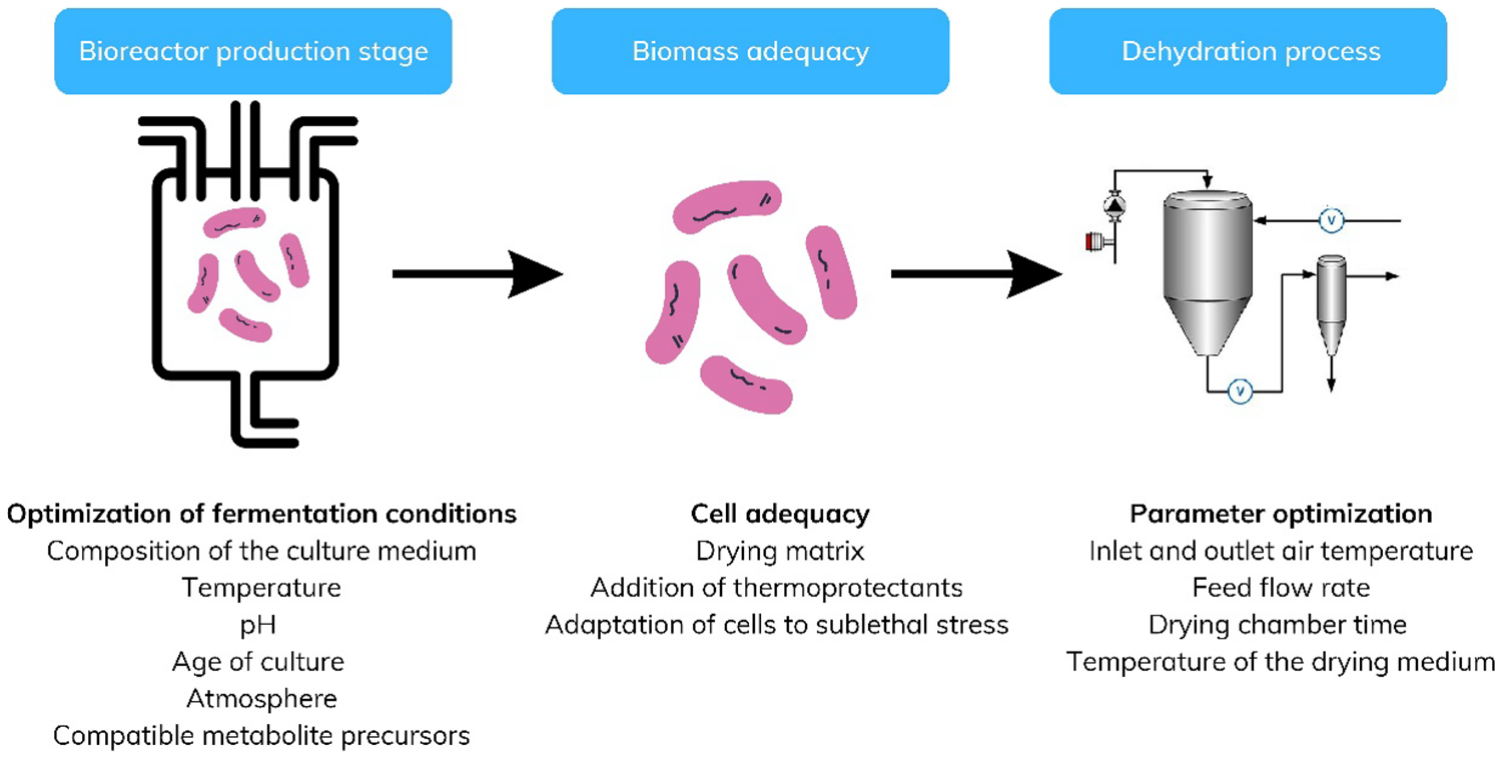
Strategies to increase cellular tolerance to stress and generate protection that contribute to reducing the negative effects of spray drying

The application of the spray-drying technique to probiotics is reduced because, during drying, the cells encounter different stress conditions that can affect their viability. These conditions include heat, osmotic, and oxidative stress; dehydration; and shear stress, a process during which particles are deformed by the action of a shearing external force during atomization [176, 177]. The heat stress and dehydration are the two main mechanisms leading to inactivation and loss of viability of probiotics [178]. However, there is a big variation between different bacterial genera and species as mentioned before [34], and therefore, monitoring on a case-by-case basis is required. It is important to maintain the probiotic properties of LAB during a bioprocess such as spray drying. The tolerance of bacteria to stress derived from the spray-drying process depends on the intrinsic tolerance of the microorganisms, the conditions of biomass production (fermentation) [152, 179], addition of protective agents, adaptation of the cells to the process parameters, and sublethal stress pre-treatments prior to drying [152]. These strategies affect the viability of probiotics immediately after drying, but also during storage. Figure 2 outlines examples of strategies to increase cellular tolerance to stress and generate protection that contribute to reducing the negative effects of spray drying.

The coating material that protects the cells from upcoming environmental stress is another crucial parameter for probiotic cell viability. Whey protein isolate (WPI) and gum arabic (GA) have been used as spray-dried encapsulation materials because of their ability to form physically strong and stable matrices [3] and also have been combined in order to increase their efficiency [180–182]. Tirta et al. reported that *Pediococcus* (*P.*) *acidilactici* cell viability was significantly affected by inlet temperature when whey protein and GA coating materials were used, but not by wall material ratios during a spray drying process. When the inlet temperature was increased to 170 °C, this caused a decrease in the viability of *P. acidilactici* by 1.36 log cycles, from 8.61 log CFU/g to 7.25 log CFU/g [3].

The drying matrix and the addition of thermoprotectants are another aspect to consider for improvement of the survival of probiotics to spray drying. These compounds show thermoprotective features, such as disaccharides (lactose, sucrose, or trehalose), dextrose, or polyols (mannitol, sorbitol), or act as probiotic growth stimulants (fructo- and galactooligosaccharides) [181–186]. An example of heat protectants to drying media (solid support) is sugars, which are frequently added in the production of dried starter cultures. They also contribute in the improvement of the survival rate of the cells during processing and further storage [187]. Oldenhof et al. [188] discussed the use of a mixture of maltodextrin and sucrose, leading to an increased survival in spray-dried lactobacilli. Sugar interacted well with lipids and proteins, and the maltodextrin functioned as an osmotically inactive loading compound leading to the formation of a glassy matrix [188]. Molecular movement is restricted by this glassy matrix and thus retards crystallization and diffusion processes, deteriorating external effects and bacterial metabolism [34]. The retail price for sucrose is approx. equal to US$ 1.45/L, used at 10% (w/v), and affecting the cost of bulk starter production. Correa Deza et al. [149, 189] formulated the thermoprotective additive containing only inorganic salts (MnSO_4_, MgSO_4_, KH_2_PO_4_, and Na_2_HPO_4_), which was successful for improvement of the cell viability of probiotic CRL-1505 strain during spray drying. These results are interesting from a technological point of view considering the price of phosphate salts (about US$ 0.40/L) and the possibility of being used as thermoprotectant during spray drying.

The induced damage can be reduced by the adaptation of LAB in physiological and metabolic aspects [77]. Mild stress adaptations including synthesis of stress protein(s), change in the fatty acid composition of the cell membrane, and transport or synthesis of compatible solutes [190] improve tolerance to multienvironmental stress by resistance of both the particular stress and other unrelated stresses [70].

### Setting of Technological Parameters

During industrial application of LAB, bacteria are subjected to various environmental stresses, hence the restriction of the growth and alteration of the physiological and biochemical properties of cells. Among the strategies that could be applied before the LAB is subjected to the dehydration process itself, we can mention protective pre-treatments to create adaptation (changes in membrane, production of stress proteins, accumulation of compatible solutes). If the previously detailed strategies are not efficient enough to achieve adequate survival, technological parameters of the dehydration process can still be adjusted. The adjustment of optimal conditions of freezing time, freezing temperature, pressure, and lyophilization time during lyophilization determines the survival of the probiotic not only at the end of the process but also during storage [117, 191].

The negative effect of the spray drying process, besides being related to intricate characteristics of the probiotic LAB, depends on the process parameters (inlet and outlet air temperature, feed flow rate, drying chamber time, drying chamber design, drying medium temperature) employed during dehydration [180, 183, 192, 193]. The use of high levels of feed flow rates can reduce heat-induced cell damage, however will result in a high water activity powder with a short shelf life [193, 194]. For most heat-sensitive strains, such as *L. acidophilus* or *L. rhamnosus* GG, an air outlet temperature between 70 and 80 °C is recommended to minimize cell injury induced by spray drying [181–183, 195].

## Postbiotics: A Strategy for Maintaining the Health-Promoting Properties of Inactivated Probiotics

Although the traditional probiotic definition presupposes that bacteria must remain alive to produce health-promoting effects, much scientific evidence has shown that formulations containing their cellular byproducts can also promote the desired response. These compounds are called postbiotics and include cell lysates (CLs), enzymes, and cell wall fragments derived from probiotic bacteria and can be an alternative to probiotics, taking into account that strain-specific behavior, antibiotic gene transfer, and the potential of some probiotic strains for infection in immunocompromised individuals could be some of the limitations of probiotics [196, 197]. When ingested in sufficient quantities, the host is provided with multiple biological health benefits [198, 199]. Some of the many postbiotic metabolites include exopolysaccharides, glycoproteins, peptides, proteins, peptidoglycans, linoleic acid, lactic acid, and short-chain fatty acids shown to have significant antioxidant, anti-inflammation, and antibacterial effects [199, 200].

Moreover, the clinical and health benefits of probiotics are not associated with viability due to the non-interdependence of the plausible mechanisms with viability as reported by Barros et al. [198]. Postbiotics possess potential health-promoting properties, and a recent study by Park et al. [199] investigated the effect of CLs of *Lactiplantibacillus plantarum* (LP CL) and *Lacticaseibacillus rhamnosus* GG (LR CL) on the inhibition of virus-mediated inflammatory responses in the human intestinal epithelial cell line HT-29 in vitro.

They found that both LP CL and LR CL could inhibit virus-mediated inflammatory responses and confer synergistic inhibitory effects with short-chain fatty acids such as butyrate in human intestinal epithelial cells. Hosseini et al. [200] studied the potential biological activities of postbiotics derived from *Saccharomyces cerevisiae* (PTCC 5269) (PSC) under in vitro circumstances. A high level of phenolics and flavonoids was detected with strong antibacterial activity against pathogens (creating eubiosis) showing that their health-promoting functions can be extended to medical, biomedical, and food scopes, for design of optimized functional food formulations or/and supplementary medications for prevention and treatment of chronic/acute disorders. Postbiotics improve the efficiency of the innate immune system, decrease the inflammatory responses caused by the presence and activity of pathogenic germs with inflammation-inducing activity and carcinogenic agents (especially those derived from food processing), and bolster the effectiveness of the intestinal barrier [204–206].

## Conclusions

Given the relationship between the microbiome and several diseases or specific symptoms, research effort should be focused on the optimal intervention of probiotics as a complementary tool for their treatment. Since the results are different between different bacterial genera and species, but also between strains of the same species, their study must be done separately for each. Further research to optimize all the conditions that contribute to dealing with stress to which probiotics are subjected and increasing their bioavailability will bring about new uses for probiotics contributing to issues concerning both the food industry and other similar industries.

## Data Availability

No datasets were generated or analyzed during the current study.

## Abbreviations

HSPs: Shock proteins
PtsI: Phosphoenolpyruvate-protein phosphotransferase
DnaK: Gene encoding the DnaK chaperone protein
ProS: Prolyl-tRNA synthetase
ProRS: Chaperone protein DnaK
GroEL: Chaperonins
Cofactors: GroES
CSPs: Cold-shock proteins
RH: Relative humidity
HDPE: High-density polyethylene
BSH: Bile salt hydrolase
BAs: Bile acids
GIT: Gastrointestinal tract
SCFA: Short-chain fatty acid
LAB: Lactic acid bacteria
*B.*: *Bacillus*
WPI: Whey protein isolate
GA: Gum arabic
*P.*: *Pediococcus*
*L.*: *Lactobacillus*, *Lacticaseibacillus*, *Ligilactobacillus*, *Lactiplantibacillus*, *Limosilactobacillus*
